# An Exploration into the Fault Diagnosis of Analog Circuits Using Enhanced Golden Eagle Optimized 1D-Convolutional Neural Network (CNN) with a Time-Frequency Domain Input and Attention Mechanism

**DOI:** 10.3390/s24020390

**Published:** 2024-01-09

**Authors:** Jiyuan Gao, Jiang Guo, Fang Yuan, Tongqiang Yi, Fangqing Zhang, Yongjie Shi, Zhaoyang Li, Yiming Ke, Yang Meng

**Affiliations:** 1 Key Lab Hydraul Machinery Transients, Wuhan University, Wuhan 430072, China; gaojiyuan@whu.edu.cn (J.G.); tongqiang.yi@whu.edu.cn (T.Y.); zfq@whu.edu.cn (F.Z.); zhouzhouzsyz@163.com (Y.S.); 2017282070272@whu.edu.cn (Z.L.); keyiming@whu.edu.cn (Y.K.); 2019302080229@whu.edu.cn (Y.M.); 2 School of Power and Mechanical Engineering, Wuhan University, Wuhan 430072, China

**Keywords:** analog circuits, fault diagnosis, improved GEO, 1D-CNN, attention mechanism

## Abstract

With the continuous operation of analog circuits, the component degradation problem gradually comes to the forefront, which may lead to problems, such as circuit performance degradation, system stability reductions, and signal quality degradation, which could be particularly evident in increasingly complex electronic systems. At the same time, due to factors, such as continuous signal transformation, the fluctuation of component parameters, and the nonlinear characteristics of components, traditional fault localization methods are still facing significant challenges when dealing with large-scale complex circuit faults. Based on this, this paper proposes a fault-diagnosis method for analog circuits using the ECWGEO algorithm, an enhanced version of the GEO algorithm, to de-optimize the 1D-CNN with an attention mechanism to handle time–frequency fusion inputs. Firstly, a typical circuit-quad op-amp dual second-order filter circuit is selected to construct a fault-simulation model, and Monte Carlo analysis is used to obtain a large number of samples as the dataset of this study. Secondly, the 1D-CNN network structure is improved for the characteristics of the analog circuits themselves, and the time–frequency domain fusion input is implemented before inputting it into the network, while the attention mechanism is introduced into the network. Thirdly, instead of relying on traditional experience for network structure determination, this paper adopts a parameter-optimization algorithm for network structure optimization and improves the GEO algorithm according to the problem characteristics, which enhances the diversity of populations in the late stage of its search and accelerates the convergence speed. Finally, experiments are designed to compare the results in different dimensions, and the final proposed structure achieved a 98.93% classification accuracy, which is better than other methods.

## 1. Introduction

Electrical equipment is widely used in nuclear power, the chemical industry, and other high-security equipment fields, and its working environment is often in high-temperature, high-pressure, high-humidity, and other harsh environments. As the system continues to operate, the reliability of its internal circuit board cards decreases due to its operating environment, which could ultimately lead to unanticipated downtime, increased economic losses, and even production accidents [1,2,3]. Furthermore, statistics have shown that although 80% of the board cards are digital, most failures occur in the analog section [4]. Therefore, the research on analog-circuit-fault-diagnosis methods has become a hot topic, which is of great importance for improving the reliability and operational efficiency of high-safety equipment, as well as efficient health management, remote operation, and digital overhaul [5].

Analog circuit faults can be classified into hard faults and soft faults based on the characteristics of the analog circuit at the time of their occurrence [6]. Hard faults are caused by short circuits or opens in components resulting in sudden changes in the circuit performance or failures, whereas soft faults are caused by analog circuit components aging over long periods resulting in parameter values outside of the allowable tolerance ranges. Hard faults can be analyzed based on the basic mechanism of the circuit to locate the faulty component and determine the type of fault, and research in this area has been intensive and has achieved a relatively mature stage [7]. In contrast, soft faults have infinite possibilities of fault values due to the continuity of component parameters and the diversity of output signals in the normal state due to the influence of component tolerances, making fault diagnosis difficult to realize, which is becoming a hot issue in current research [8,9,10]. C. Alippi et al. [11] proposed a harmonic analysis-based method for fault diagnosis in analog electronic circuits, using a “simulation before test” approach. Through randomized algorithms, the method efficiently creates a fault dictionary and selects optimal test inputs, applicable to various circuit complexities. Y. Deng et al. [12] proposed a new method for locating parametric faults in nonlinear analog circuits using subband decomposition and coherence functions. By analyzing the Volterra series with wavelet packets and comparing them with fault signatures, the method accurately identifies and locates faults, as demonstrated by simulations. Although fault-diagnosis methods based on prior knowledge and mechanism analysis can yield effective results, the increasing complexity of electronic systems presents new challenges. Analog circuits are inherently highly nonlinear, with components that have varying tolerances and are susceptible to noise interference. These characteristics significantly impact the effectiveness of traditional machine learning methods, which alone are insufficient to meet the requirements of current analog circuit fault-diagnosis tasks.

The emergence of deep learning has brought new possibilities and perspectives to analog circuit fault diagnosis, providing new tools and methods to tackle various challenges in complex electronic systems and analog circuits through its powerful data-processing and automatic feature-extraction capabilities. H. Jung et al. [13] proposed an algorithm that employs frequency analysis and artificial intelligence, specifically convolutional neural networks (CNNs), to detect failures in industrial rotors. By converting rotor fault sounds into spectrograms and analyzing them with CNNs, the system achieves over 99% accuracy in distinguishing between normal and faulty states. G. Puvaneswari et al. [7] proposed a faster regional-convolutional-neural-network-based method for test node selection in complex analog circuits, optimizing fault dictionary techniques. Simulations consider both hard and soft faults, and the proposed method achieves a computational time of 0.2 s and an accuracy rate of 97.4%, outperforming other techniques in various metrics. J. Yang et al. [14] proposed an attention-weighted graph convolution network (Att-GCN) method for diagnosing incipient faults in analog circuits, which are challenging due to subtle fault features. The Att-GCN, combining spatial-domain graph convolution with an improved self-attention mechanism, extracts comprehensive fault features. A multisample dropout method prevents overfitting. Testing on various circuits, the method shows improved accuracy in detecting incipient faults in analog systems.

Due to the characteristics of the soft fault itself, when processing the output signals of each fault state and normal state, the extracted fault characteristics are prone to overlapping, which makes it difficult to locate the location of the faulty component. Therefore, traditional methods that rely on experience to determine network structure are no longer applicable. Employing parameter-optimization algorithms to determine the optimal structure of the model can effectively enhance the feature-extraction capability of the machine learning model. Y. Li et al. [15] proposed a novel CNN model, named GA-CNN, for hyperspectral image (HSI) classification. Tested on four HSI datasets, the GA-CNN surpasses conventional CNNs in classification accuracy with fewer parameters. W. Lu et al. [16] proposed a hybrid model named GA-CNN-LSTM to predict daily tourist flow at China’s Huangshan Scenic Spot, recognizing the challenges posed by the nonlinear characteristics of tourist flow data. Compared to other models, like CNN-LSTM, LSTM, CNN, and BPNN, GA-CNN-LSTM outperforms, with an MAPE improvement of approximately 8.22% over CNN-LSTM.

**Figure 1:**
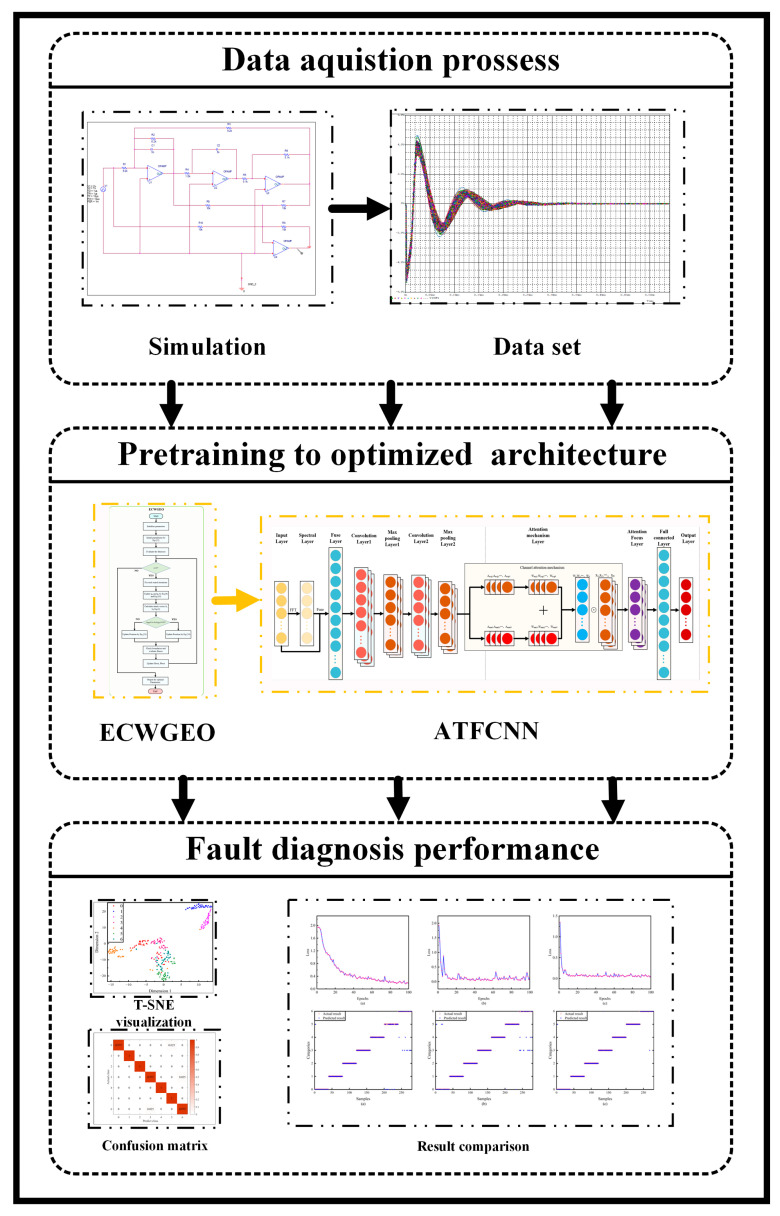
Fault diagnosis flowchart.

In this paper, a novel intelligent fault-diagnosis method is proposed to address the aforementioned challenges in analog circuit fault diagnosis, as illustrated in Figure 1, with the main steps being as follows:

(1) A Pspice model was built for typical analog circuits, and a variety of fault injections closer to the real situation were carried out, experimental data were obtained using Monte Carlo analysis, and the training and test sets were divided.
(2) The fault injection method is closer to the actual situation, which leads to the aggravation of the fault features’ overlapping, which brings more challenges to the fault feature extraction. To solve this problem, the proposed method does not rely on experience to design the network structure, but selects the GEO algorithm and improves it according to the characteristics of deep learning network structure design, adding a chaos operator to solve the problem of decreasing population diversity in the late stage of optimization, as well as strengthening the search strategy to enhance the convergence speed and is named ECWGEO; the results show that the optimized algorithm has a certain advantage in both the convergence speed and the selection of the final results. Result selection has certain advantages.
(3) To further explore the potential value of the data and enhance the fault-diagnosis ability of the model, the one-dimensional convolutional algorithm was improved by adopting the joint input mechanism in the time–frequency domain and adding the attention mechanism. The results show that the joint input mechanism provides richer features for the network, the existence of the attention mechanism improves the feature-selection ability of the model, and the proposed network achieves good results.

The organization of the paper is as follows. The second part of this thesis describes the methodology used to construct the model, which includes the GEO and attention mechanism. The third part explains the proposed method, detailing the improvements made to both the GEO and network structure. In the fourth part, the process of building the simulation model and acquiring the dataset is described. The fifth part consists of evaluating and comparing the constructed models. Finally, the conclusion is presented in the last part.

## 2. Related Research

In this section, this article focuses on some algorithmic models and optimization methods related to the proposed fault-diagnosis model.

### 2.1. Golden Eagle Optimizer

**Figure 2:**
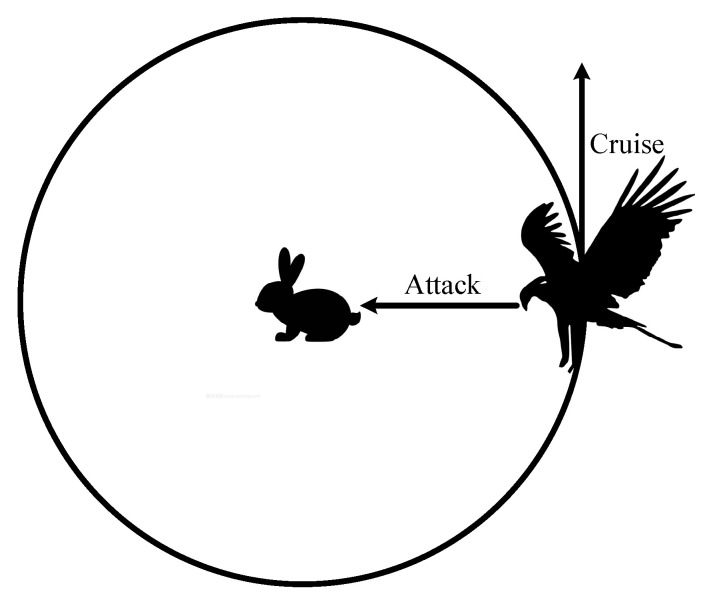
Spiral motion of golden eagles.

The Golden Eagle Optimizer (GEO) is a new meta-heuristic algorithm proposed by Mohammadi-Balani et al. [17]. The algorithm performs a parameter search by simulating learning the behavior of golden eagle predation in nature. The GEO consists of three parts: parameter initialization, exploration, and exploitation. Exploration and exploitation are implemented using attack vectors and cruise vectors [18,19,20]. Figure 2 is the schematic diagram of the attack and cruise vectors of the golden eagle in the 2D case.

#### 2.1.1. Parameter Initialization

The GEO begins with random initialization, like the majority of population-based optimization algorithms. The population initialization for mongoose candidate solutions is X in Equation (1), as specified in the first step in the GEO. This population is produced randomly between the lower and upper bounds of a particular problem.

(1) $$X=\left[\begin{array}{ccc} x_{1,1} & \cdots & x_{1,d}\\ \vdots & x_{i,j} & \vdots \\ x_{n,1} & \cdots & x_{n,d} \end{array}\right],$$

X is the set of current candidate populations, which are created at random using Equation (2), $X_{i,j}$ stands for the position of the jth dimension of the ith population, n stands for population size, and d stands for the dimension of the problem.

(2) $$X_{i,j}=\mathrm{unifrnd}(\mathrm{VarMin},\mathrm{VarMax},\mathrm{VarSize}),$$

VarMin and VarMax are the problem’s lower and upper bounds, respectively, and unifrnd is a random number with a uniform distribution. VarSize refers to the problem’s dimensions or the decision variables’ size. The best solution obtained in each iteration is the best solution obtained thus far.

#### 2.1.2. Exploitation and Exploration

The attack process of the golden eagle is described by Equation (3), where $A_{i}$ is the attack vector of the ith eagle, $X_{f}^{*}$ is the optimal solution currently found by golden eagle f, and $X_{i}$ is the current position of golden eagle i.

(3) $$A_{i}=X_{f}^{*}-X_{i},$$

The cruise vector is the tangent vector to the circle, perpendicular to the attack vector. To compute the cruise vector, one must first compute the equation of the tangent hyperplane. Equation (4) represents the scalar form of the hyperplane equation in n-dimensional space.

(4) $$h_{1}x_{1}+h_{2}x_{2}+\cdots \;+x_{n}h_{n}=d\Rightarrow \sum_{j=1}^{n}h_{j}x_{j}=d$$

where $\vec{H}=[h_{1},h_{2},\cdots ,h_{n}]$ is the normal vector and the hyperplane in which the cruise vector is located and can be computed using Equation (5).

(5) $$\sum_{j=1}^{n}a_{j}x_{j}=\sum_{j=1}^{n}a_{j}^{t}x_{j}^{*}$$

where ${\vec{A}}_{i}=[a_{1},a_{2},\cdots ,a_{n}]$ is the attack vector, X = $\left[x_{1},x_{2},\cdots ,x_{n}\right]$ , and $X^{*}$ = $\left[x_{1}^{*},x_{2}^{*},\cdots ,x_{n}^{*}\right]$ is the position of the selected predator. In GEO, the index of the selected fixed vector is denoted by y. Meanwhile, the remaining n − 1 free vectors are assigned random values. The value of the fixed variable is then determined using Equation (6).

(6) $$c_{y}=\frac{d-\sum_{j,j\neq y}b_{j}}{b_{y}}$$

among others, $c_{y}$ is the yth element of $c_{i}$ the yth element of b_j_, and b_y_ are the jth and yth elements of A_i_ the jth and yth elements of D. D is the value of the right-hand side of Equation (3). Once the fixed variables of the cruise hyperplane are established, the cruise vector is defined by Equation (7).

(7) $$C_{i}=\left(c_{1}=\;\mathrm{random}\;,c_{2}=\;\mathrm{random}\;,\ldots ,c_{y}=\frac{d-\sum_{j,j\neq y}b_{j}}{b_{y}},\ldots ,c_{n}=\;\mathrm{random}\;\right),$$

#### 2.1.3. Move to a New Position

The position update of the golden eagle is determined based on the attacking behavior and the cruising behavior. The step size of the two behaviors of the ith golden eagle in iteration t is denoted as Equation (8), where r_1_ and r_2_ are hyperparameters in the range of 0 to 1, and the $p_{a}^{t}$ and $p_{c}^{t}$ are calculated using Equations (9) and (10), which are the coefficients of the attack vector and the coefficients of the cruise vector in iteration t. T is the maximum number of iterations. $∥A_{i}∥\;\mathrm{and}\;∥C_{i}∥$ are the Euclidean norms of the attack vector and the cruise vector, computed based on Equations (11) and (12).

(8) $$\Delta x_{i}^{t}=r_{1}p_{a}^{t}\frac{A_{i}}{∥A_{i}∥}+r_{2}p_{c}^{t}\frac{C_{i}}{∥C_{i}∥}$$

(9) $$p_{a}^{t}=p_{a}^{0}+\frac{t}{T}\left|p_{a}^{T}-p_{a}^{0}\right|$$

(10) $$p_{c}^{t}=p_{c}^{0}-\frac{t}{T}\left|p_{c}^{T}-p_{c}^{0}\right|$$

(11) $$∥A_{i}∥=\sqrt{\sum_{j=1}^{n}a_{j}^{2}}$$

(12) $$∥C_{i}∥=\sqrt{\sum_{j=1}^{n}c_{j}^{2}},$$

As Equation (13) shows, the position of golden eagle i in round t + 1 is its position in round t plus the step vector in round t.

(13) $$x_{i}^{t+1}=x_{i}^{t}+\Delta x_{i}^{t}$$

### 2.2. Related Works on the GEO

In response to the evolving needs and challenges in the field of the parameter search, a substantial body of work has been dedicated to enhancing the standard GEO algorithm. This section delves into the pivotal contributions and methodologies that have shaped the current landscape of GEO algorithm improvements.

Fan et al. [21] have enhanced the position update mechanism of the traditional GEO. Their improvement addresses the slowing down of the algorithm’s convergence speed when nearing the global optimum and its inefficiency in multimodal functions. They introduced a new mechanism called “Stooping” behavior. This approach draws inspiration from the way eagles dive rapidly and powerfully during hunting. It simulates this stooping action in the algorithm’s position-updating phase. By incorporating this behavior into a mathematical model, the strategy effectively speeds up the algorithm’s convergence when it is close to finding the global optimum. It also ensures that the algorithm retains its ability to explore effectively. Under this new strategy, the algorithm switches from its traditional update mode to the Stooping mode. This switch occurs when certain conditions are fulfilled, such as the algorithm getting close to its prey or reaching a predetermined number of iterations.

Panneerselvam et al. [22] focused on enhancing the global search capabilities of algorithms. They identified a key limitation in the traditional Golden Eagle Algorithm: a lack of flexibility and adaptability in exploring and exploiting the search space. This limitation resulted in reduced efficiency in finding global optima. To address this, they made improvements to the GEO. These improvements involved introducing new adaptive features. These features significantly enhance the algorithm’s efficiency in navigating unknown search spaces. The adaptive nature of these features allows the algorithm to dynamically adjust its strategies. This adjustment is based on the current state of the search. It leads to an improved balance between exploration, which is the global search, and exploitation, which is the local search.

Deng et al. [23] made significant improvements to the traditional GEO algorithm. In the process of population initialization, they introduced the Arnold Chaotic Map to generate a more diversified initial position, thereby enhancing the global search capability of the algorithm. To better balance the abilities of exploration and exploitation, a nonlinear convex decreasing weight was incorporated. This adjustment enables the algorithm to modify its search behavior according to different phases. Additionally, they revised the position update formula of the GEO, integrating a global optimization strategy. In each iteration, the best-performing individual is selected for the interaction, thus improving the algorithm’s efficiency in solving complex optimization problems.

In response to specific needs across different application domains of the GEO algorithm, numerous researchers have implemented targeted modifications and optimizations, resulting in enhanced performance in various aspects. Given the characteristics of the optimization problem addressed in this paper, we aim to increase the convergence speed of the algorithm and seek solutions that are more aptly suited to these issues. Detailed descriptions of these improvements and their impacts on optimization performance will be elaborated on in the subsequent sections.

### 2.3. Attention Mechanism

**Figure 3:**
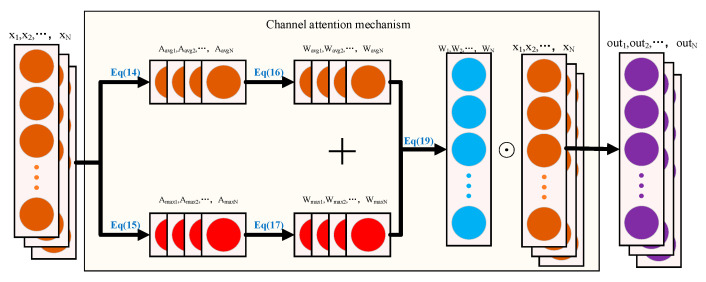
Schematic diagram of the channel attention mechanism.

In a one-dimensional convolutional neural network (1D-CNN), each feature channel is usually considered to be of equal importance, although the importance of the information they carry may differ. This equal treatment of channel importance lacks rationality. Therefore, the SEnet proposed by Hu et al. [24] provides a way to adaptively recalibrate channel feature responses by explicitly modeling the dependencies between channels. Each feature channel can be regarded as a specialized detector in this architecture, and the importance of each channel is evaluated through compression and excitation operations, which enable an explicit correlation of the relationships between different feature channels. Figure 3 is a schematic representation of the attention mechanism.

First, the input tensor is compressed using global average pooling and global maximum pooling; see Equations (14) and (15). N is the length of the single-channel data, and the A_avg_ and x_i_ are the compressed eigenvalues and the data of the ith channel [25].

(14) $$A_{avg}=\frac{1}{N}\sum_{i=1}^{N}x_{i}$$

(15) $$A_{\max}=\max_{i=1}^{N}x_{i},$$

Subsequently, both A_avg_ and A_max_ are fed into the fully connected layer FC for processing, e.g., Equations (16) and (17).

(16) $$W_{avg}=FC2\left(FC1\left(A_{avg}\right)\right)$$

(17) $$W_{\max\;}=FC4\left(FC3\left(A_{\max\;}\right)\right),$$

The two weights obtained are summed and passed through a sigmoid activation function, which is subsequently multiplied by the original input tensor to obtain the tensor Output, which is then fed into the next layer of the model [26].

(18) $$W_{\mathrm{combined}\;}=W_{\mathrm{avg}\;}+W_{\max\;}$$

(19) $$W=\mathrm{sigmoid}(W_{\mathrm{combined}\;})$$

Output = W ⊙ x(20)

## 3. The Proposed Method

In this section, we will detail how innovative modifications have been made to the GEO algorithm, as well as the CNN algorithm.

### 3.1. Enhanced Chaos-Weighted GEO (ECWGEO)

**Figure 4:**
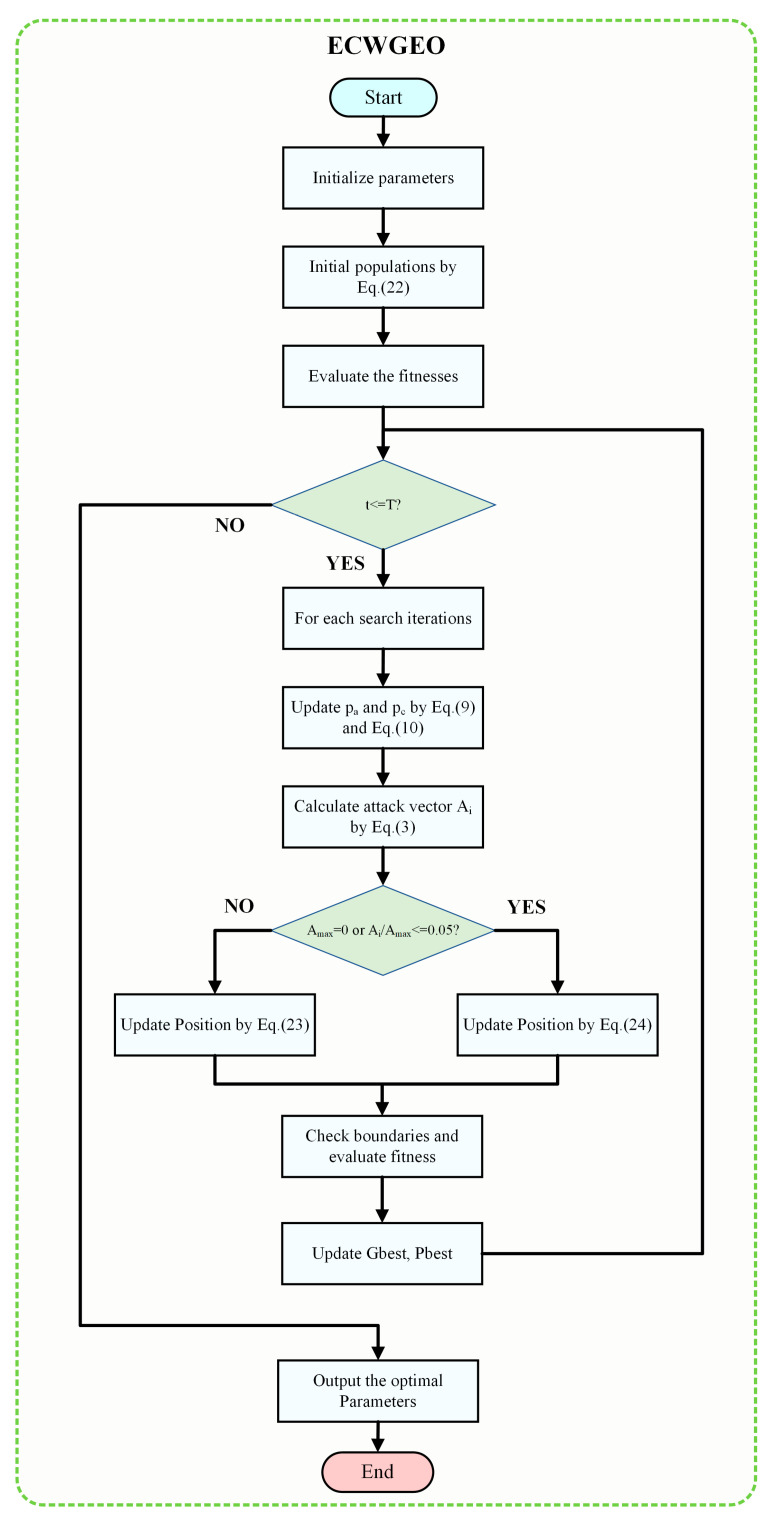
The flowchart of the proposed ECWGEO.

The traditional GEO suffers from two main shortcomings: a lack of population diversity and slow convergence. These problems are particularly evident in application scenarios used for network structure optimization in the pre-training phase. A lack of population diversity directly affects the optimization of the network structure, which reduces the accuracy of the fault diagnosis model, while the low convergence speed significantly increases the computational burden. To solve the above problems, the ECWGEO incorporates chaos operators and enhanced predation strategies. Figure 4 is the flow chart of the ECWGEO algorithm.

In the standard Golden Eagle Optimization (GEO) algorithm, the positions of golden eagles are updated by simulating their natural behaviors (e.g., observing, chasing, and feeding). As the number of iterations increases, the golden eagles may cluster around one or a few locally optimal solutions, which reduces the population diversity and hence the ability of the algorithm to jump out of the local optimal solutions; and since the update formula depends on the distance when most golden eagles are close to the globally optimal solution, the search space will become finite, resulting in the algorithm having difficulty in finding a better solution again. Inspired by the literature [27,28,29], the article introduces a chaotic weighting factor that generates random and unordered values between −1 and 1, and it is defined as Equation (21). This chaotic weight operator is introduced to the process of initialization of the golden eagle population and the process of the global parameter search. Equation (22) describes the process of parameter initialization; for each golden eagle i and each dimension j, the initial position X_ij_ can be updated by introducing the chaos operator map_ij_ Equation (22) after the re-updating of the equation. The new position x^t^*^+^*^1^ is updated using Equation (23).

(21) $${\mathrm{map}}_{t+1}=\sin\left(\frac{c\times \pi }{{\mathrm{map}}_{t}}\right),{\mathrm{map}}_{1}=0.7$$

(22) $$X_{\mathrm{ij}}=\mathrm{VarMin}+\left(\mathrm{VarMax}-\mathrm{VarMin}\right)\times \frac{{\mathrm{map}}_{\mathrm{ij}}+1}{2}$$

(23) $$x^{t+1}=x^{t}+{\mathrm{map}}_{t}\Delta x_{i}^{t},$$

**Figure 5:**
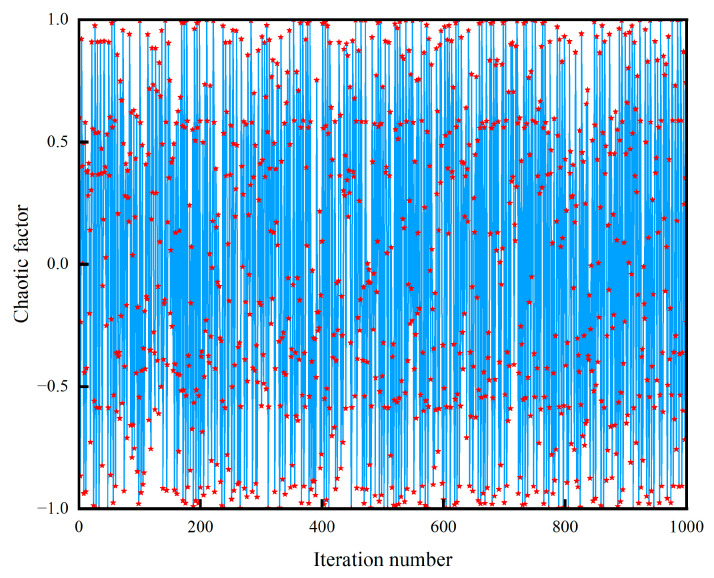
Chaotic behavior across iterations.

Figure 5 demonstrates that the chaos operator has strong fluctuation and randomness. In the image, the stars represent the values of the chaos operator ‘ ${\mathrm{map}}_{t}$ ’ at iteration ‘t’. The use of its nonlinear property for position initialization can significantly improve the randomness of the initial population. At the same time, as a perturbation factor for position updating, it can increase the diversity of the population at the later stage of iteration, so that the population does not easily fall into the local optimum.

In the standard GEO, the local search is mainly based on updating the position based on the distance between each golden eagle and the current optimal solution. This mechanism could be effective for the global search in the initial stages. However, it can be particularly detrimental when applied to computationally complex scenarios, such as deep learning structure optimization requiring fast convergence, which is a result of the slow convergence rate caused by the greatly increasing amount of computation. To address this challenge, this paper draws on the ideas of the literature [30,31] and designs a new position update formula Equation (24), which strengthens the local search capability of the algorithm and significantly enhances the convergence speed of the algorithm.

(24) $$X=\mathrm{Gbest}\;+\mathrm{mu}\times \left(\left(\mathrm{Varmax}-\mathrm{Varmin}\right)\times \;\mathrm{rand}+\mathrm{Varmin}\right)\times \mathrm{Dir}$$

(25) Dir=sgn(rand − 0.5)

(26) $$mu=\gamma \times e^{\left(-{\left(\alpha \times \frac{t}{T}\right)}^{\beta }\right)},$$

where Dir controls the direction of search, mu is a nonlinear parameter, t is the current number of generations, T is the maximum number of search generations, and γ, α, and β are three constants used to control the algorithm exploration and exploitation capabilities, and in this study, after empirical testing, we set the values of the parameters as γ=3 , α=3.5 , and β=2.5 .

### 3.2. Attention Time–Frequency Convolution Neural Network (ATFCNN)

To solve the problem of fault feature aliasing due to the characteristics of analog circuits themselves, such as high nonlinearization, component tolerance, and susceptibility to noise interference, this article makes some improvements to the CNN and proposes the ATFCNN algorithm.

**Figure 6:**
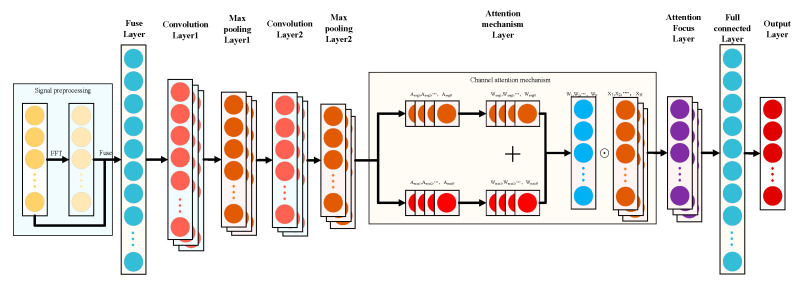
The structure of ATFCNN.

The overall ATFCNN is shown in Figure 6. The algorithm consists of a time–frequency conversion layer, a convolutional layer, a pooling layer, a channel attention layer, a fully connected layer, and a dropout layer. Before the convolution operation, the time-domain signal is preprocessed, the frequency-domain features are obtained by FFT, and then, the time–frequency input signal obtained based on fusion with the time-domain signal can contain richer features, which effectively enriches the information captured by the model, and the attention mechanism is added to the subsequent network structure, which makes the more important features have greater weight and the secondary information have a lower weight, to improve the classification accuracy.

The raw voltage signal is preprocessed before it is fed into the convolutional part. The input voltage signal is subjected to a Fast Fourier Transform to extract key features in the frequency domain, capturing periodicity and other non-temporal properties of the voltage signal. The original time-domain signal is then spliced with its frequency-domain representation to generate a fused feature layer, which serves as the input to the convolutional layer.

The convolutional part consists of two consecutive convolutions and a maximum pooling operation. Key parameters, such as the size and number of convolution kernels, are automatically determined based on a parameter-optimization algorithm. For the selection of the activation function, we chose ReLU which ensures effective gradient propagation of the network in the deep layers, thus preventing the problem of gradient vanishing and accelerating the convergence of the model, due to its linear non-saturation.

The pooled feature maps are fed into the attention mechanism layer for processing and subsequently fed into a fully connected layer, which integrates these features into a final output for fault diagnosis. The central goal of this book is to highlight the features that are most useful for fault diagnosis by dynamically assigning different weights to each channel and to reduce the influence of other less relevant features.

## 4. Experiment

**Figure 7:**
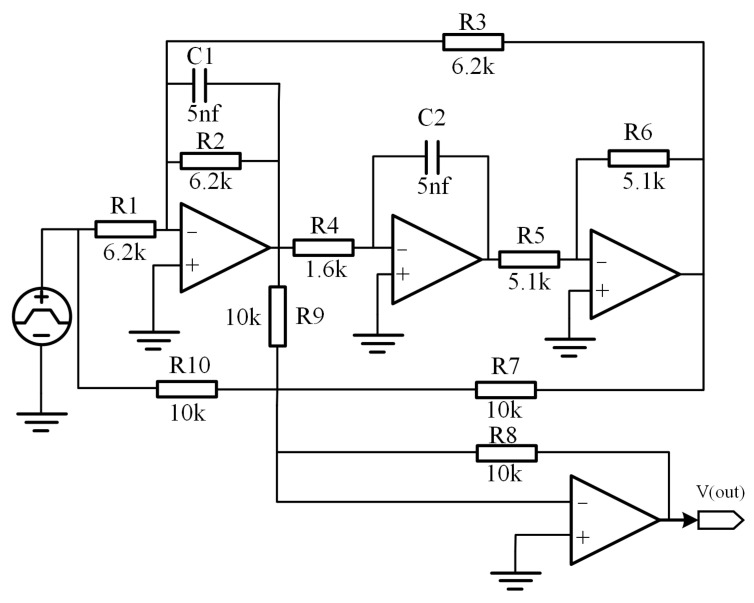
Schematic for the four-op-amp biquadratic filter circuit.

To verify the effectiveness of the proposed ECWGEO-ATFCNN for solving analog circuit troubleshooting, this paper adopts the four-op-amp biquadratic filter circuit, a highly common and representative circuit in the field of analog circuit fault diagnosis [14,32], as shown in Figure 7.

In the daily use of the circuit board, the capacitor may be degraded by the dielectrics after many charge/discharge cycles, resulting in a decrease in the capacitance value and a decrease in resistance due to soldering. The capacitance value decreases while the resistance may increase due to poor soldering or oxidation of the soldering points, resulting in an overall increase in the resistance value. In addition, the prolonged operating temperature and humidity, mechanical stress, or external environmental factors may also lead to changes in component parameters. Therefore, the sensitivity analysis of the circuit leads to the selection of C1, C2, R1, R2, R3, and R4 as the faulty components. Accurate fault simulations and injections based on actual board operating scenarios have been performed, as Table 1 shows.

**Figure 8:**
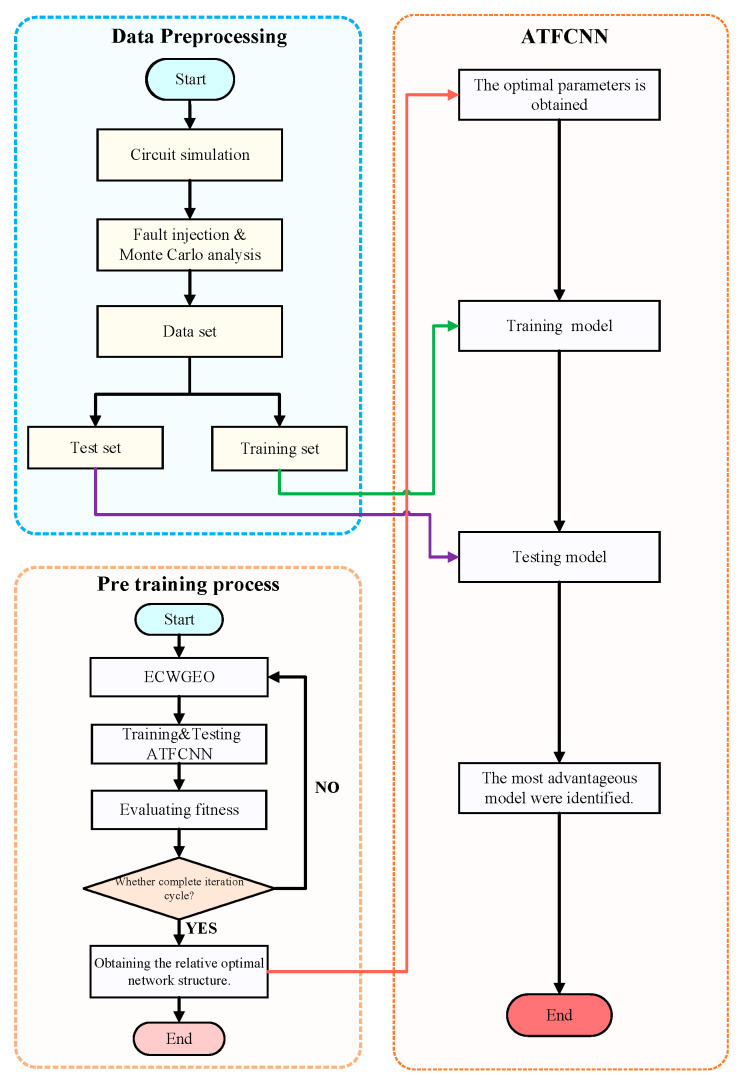
The flowchart of the proposed method.

The flow of the proposed ECWGEO-ATFCNN model is shown in Figure 8.

Simulation experiments for the former circuit were completed in PSPICE(16.6). The tolerance of resistance and capacitance were set to 5% and 10%. The circuit was given a 10 us 5 V square wave excitation signal, the sampling time was set to 1 ms, the sampling interval was set to 1 us, and 200 Monte Carlo analyses were performed for each fault category. To avoid biased models due to data imbalance, 80% of the data was randomly selected for training and 20% of the data was randomly selected for testing for each category. The data were randomly selected for testing.

After completing the training set partitioning, the model enters the pre-training phase, and the main parameters of ECWGEO are set as follows: the number of filters in the first convolutional layer (16–64), the number of filters in the second convolutional layer (64–256), the size of the convolutional kernel (2–8), the learning rate (0.0001–0.1) and dropout rate (0–0.5), and the population size is set to 30 and maximum iteration is set to 100. Twenty epochs are used for model training, the batch size is 32, and the fitness function is set as shown in Equation (27).

(27) $$\mathrm{Fit}=\frac{1}{N}\sum_{p=1}^{N}\sqrt{{\left(P_{po_{1}}-T_{po_{1}}\right)}^{2}+{\left(P_{po_{2}}-T_{po_{2}}\right)}^{2}+\ldots +{\left(P_{po_{7}}-T_{po_{7}}\right)}^{2}},$$

In the above equation, M represents the number of samples; $P_{po_{x}}$ and $T_{po_{x}}$ represent the predicted output value and expected output value, respectively, of the pth sample in the 1, 2 ⋯7 neurons of the output layer.

After the pre-training completes the network structure optimization, the model enters the formal training phase; the model training uses 100 epochs, the batch size is set to 32, and the network structure takes the optimal structure found based on the pre-training.

## 5. Result

The results of the experiments, as well as the comparisons, are shown and discussed in detail in this section, which contains four parts: parameter optimization validation, network structure validation, small sample validation, and a comparison of the rest of the algorithms, which demonstrate the effectiveness of the proposed algorithms in the comparison with each other from multiple dimensions.

### 5.1. Parameter Optimization Validation

In this section, the main focus will be to verify the effectiveness of the proposed parameter-optimization algorithm.

**Figure 9:**
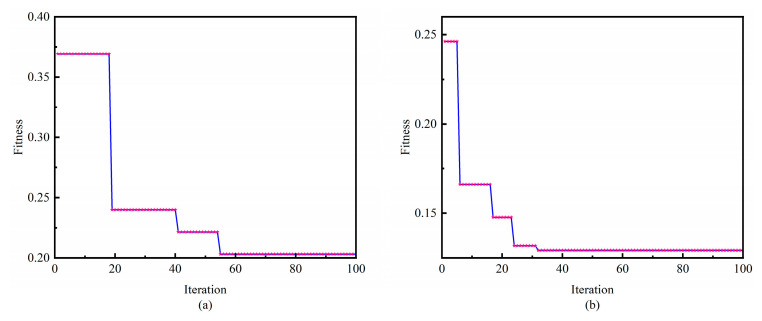
Comparative Analysis of Parameter optimization. (**a**) GEO, (**b**) ECWGEO.

Figure 9 shows the process of network structure optimization in the pre-training phase of the GEO and ECWGEO. It can be seen that compared with the traditional GEO, which completes convergence in 55 generations, the ECWGEO completes convergence 23 generations earlier, and the value of the fitness is reduced by about 36.63%, which can be reflected by the fact that the addition of the enhanced predation strategy makes the algorithm explore the local solution space in a more in-depth and efficient manner, which speeds up the algorithm’s convergence speed and finds a better solution. The results of parameter optimization are shown inTable 2. To verify the effectiveness, we also compared it with the ATFCNN model with empirical parameters set.

**Figure 10:**
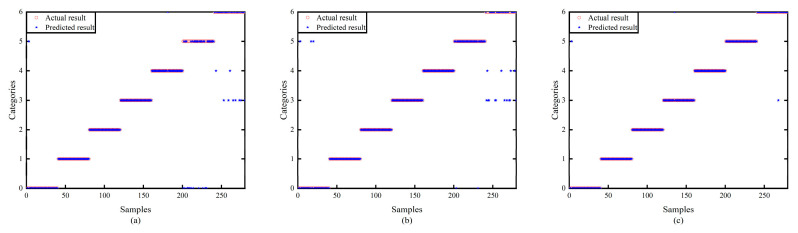
Classification results of models. (**a**) Standard ATFCNN Parameter, (**b**) ATFCNN with GEO Optimization, (**c**) ATFCNN with ECWGEO Optimization.

Figure 10 shows the classification effect under different network structures. The accuracy of ATFCNN with (a) as the standard parameter is only 91.07%, and the classification result of ATFCNN with GEO optimization is shown in (b), which obtains a certain effect enhancement and reaches an accuracy of 93.21%. After ECWGEO optimization a very good classification result is achieved, as shown in (c), reaching a 98.93% correct classification rate, with only three sets of data being misclassified. This is because the ECWGEO has a more efficient and accurate parameter search capability and successfully locates a network structure that is more suitable for this task. This structure can dig deeper into the feature differences between categories, thus achieving a significant improvement in classification accuracy.

### 5.2. Network Structure Validation

In this section, the superiority of the proposed network structure will be verified.

**Figure 11:**
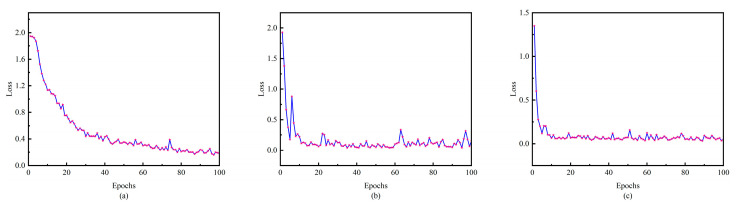
Different network performances. (**a**) CNN, (**b**) time–frequency domain CNN, (**c**) ATFCNN.

**Figure 12:**
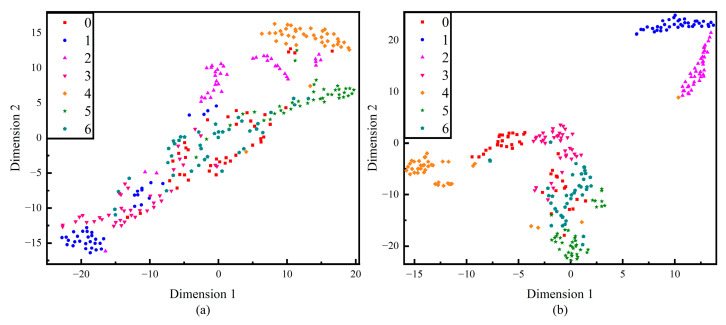
Comparative t-SNE visualization. (**a**) before the attention mechanism layer, (**b**) after the attention mechanism layer.

**Figure 13:**
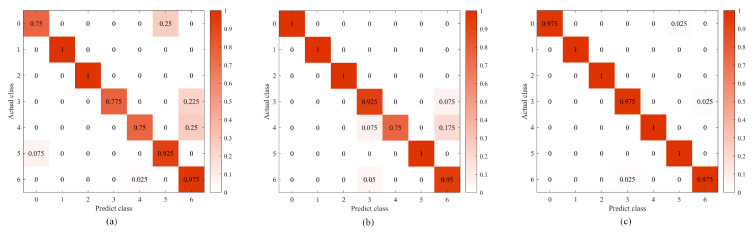
Confusion matrix of different neural networks. (**a**) CNN, (**b**) time–frequency domain CNN, (**c**) ATFCNN.

To verify the superiority of the proposed network structure, the proposed method is compared with CNN and the time–frequency domain CNN. As Figure 11 shows, in the early stages of training, (b) and (c) converge much faster than (a) in the initial training phase, which proves that the time–frequency domain splicing provides the network with rich information and helps it capture the data characteristics more easily. Comparing the average loss, (c) shows a decrease in 42.54% compared to (b), which is attributed to the addition of the attention mechanism, which enables the model to process the key information more accurately and to visualize the effect of the attention mechanism; Figure 12 is a visualization of the t-SNE dimensionality reduction of the layers before and after the attention mechanism layer, and it can be seen that the addition of the attention mechanism has significantly improved the situation of multi-category aliasing, and only category 6, category 2, and category 0 are still aliased. Figure 13 shows the confusion matrices of the three network structures, and the accuracy of this method is as high as 98.93% compared to 88.21% for 1D-CNN and 94.64% for the time–frequency domain CNN.

### 5.3. Small Sample Validation

**Figure 14:**
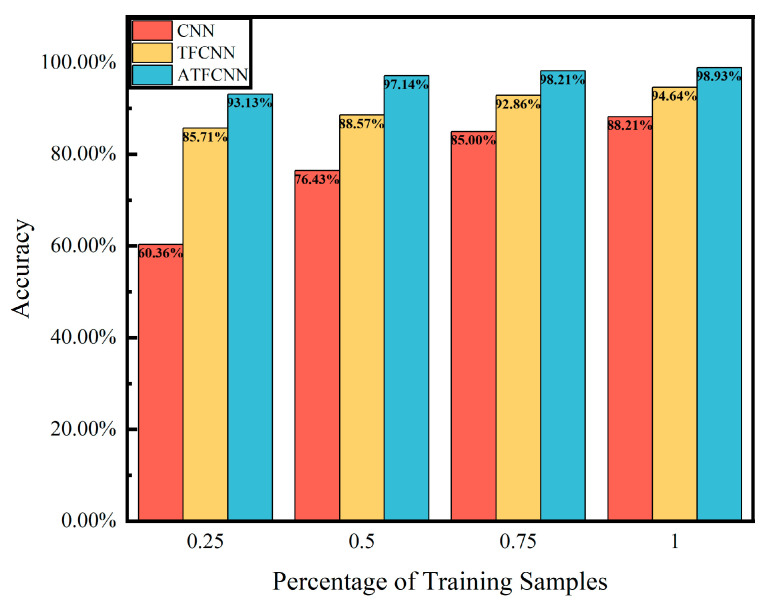
Performance under varying sample sizes.

This section aims to verify the performance of the proposed fault diagnosis model in small-sample scenarios. This further demonstrates the robustness and efficiency of the method. The model is particularly advantageous in practical applications where fault data resources are limited. Figure 14 shows the performance of the network when the test set is unchanged and the training set is changed to 25%, 50%, 75%, and 100% of the original samples. When the amount of data is changed to 25%, the accuracy of the CNN model is 60.36%, TFCNN reaches 85.71%, and the method proposed in this paper reaches 93.13%. This indicates that CNN makes it difficult to capture key features when facing limited data, while the splicing of time–frequency domain data and the addition of the attention mechanism significantly enhance the feature-extraction ability to accurately locate and process the key features, thus obtaining a higher accuracy rate under the same data conditions. As the amount of data increases, the accuracy of all three models improves, with a significant growth rate for the CNN and a slow growth rate for the remaining two models, and when the amount of data exceeds 75%, the growth of accuracy for all three models becomes slow, which means that with the support of enough data, the models begin to approach the upper limit of performance in that state, but the method in this paper still maintains a significant advantage.

### 5.4. Comparison of the Rest of the Algorithms

To further verify the effectiveness of the ECWGEO-ATFCNN fault diagnosis model, this paper selects GEO, particle swarm algorithm [33] (PSO), quantum particle swarm algorithm [34] (QPSO), and blue whale optimization algorithm [35] (WOA) for the comparison. In the pre-training stage for adaptation evaluation, 20 epochs are trained, the algorithm is trained for 100 iterations, and the network adopts the adaptation result as the evaluation index. Since the algorithm has a certain degree of randomness, this paper conducted three experimental validations, and the results of the best, worst, and average accuracy are listed. Table 3 shows the performance of each arithmetic optimization algorithm; the ECWGEO algorithm performs the best in all the tests, with a best fitness of 0.12925, and its average and worst fitness is better than the other algorithms. QPSO performs relatively poorly with the lowest fitness of 0.31562. The other three algorithms perform in between, while ECWGEO’s fitness remains relatively stable in all three runs, with QPSO’s variation being the largest. In summary, the ECWGEO shows the best performance in the task of network-structure optimization.

To further validate the effectiveness of the ECWGEO-ATFCNN fault-diagnosis model, this paper selected ATFCNN, CNN, Back-Propagation neural network [36] (BPNN), and Recurrent neural network [37] (RNN) algorithms for the comparison. The algorithms were trained for 100 epochs, and each algorithm was trained 35 times. Table 4 shows that ATFCNN is ahead of the other three algorithms. It achieves the best performance of 98.93%, and none of the other three algorithms’ best performance exceeds 91%. It is significantly better than the other three algorithms. The best classification accuracy of BPNN exceeds that of CNN and RNN, but its average and worst performance is not much different from the other algorithms. The accuracy of RNN and CNN is similar, but RNN has a slight advantage in the worst case. The performance fluctuation range of ATFCNN is 1.07%, while the fluctuation ranges of RNN, CNN, and BPNN are 2.5%, 1.42%, and 5.35%, respectively, indicating that ATFCNN is relatively more stable.

## 6. Conclusions

In this paper, an analog-circuit-fault-diagnosis method based on ECWGEO-ATFCNN is proposed, which takes into account the signal time–frequency domain feature information and achieves the network structure preference and the circuit signal fault state identification through the improved GEO algorithm and 1D-CNN fusion of the channel attention mechanism, and the main conclusions are as follows:

1. In this study, an improved GEO algorithm is proposed to optimize the network parameters in the pre-training stage of the 1D-CNN. The algorithm overcomes the problem of insufficient population diversity in the late iteration of the traditional GEO algorithm, and the chaos operator is used as a perturbation factor to improve the population diversity. At the same time, to accelerate the convergence speed of the algorithm, a strengthened search strategy is added based on the above for position updating, which effectively improves the convergence speed of the algorithm and reduces the amount of computation.
2. In this study, an improved 1D-CNN network structure incorporating the channel attention mechanism is proposed. Considering that analog circuit signals contain rich feature information in both time and frequency domains, the network fuses time-domain signals and frequency-domain signals as network inputs. At the same time, due to the different importance of information carried by different channels in the 1D-CNN network, this paper introduces the channel attention mechanism to dynamically fuse multi-channel feature information. The algorithm can extract fault feature information more effectively and comprehensively and improve the network diagnosis performance.
3. This paper takes the widely used four-op-amp biquadratic filter circuit in analog circuit fault diagnosis as the research object. It makes a reasoned selection of fault values and performs fault injection based on actual circuit component fault patterns, enhancing the practical utility of the fault-diagnosis method.
4. To verify the effectiveness of the ECWGEO-ATFCNN algorithm, this paper designs the verification experiments from the aspects of parameter optimization, the network structure, small samples, and an algorithm comparison, respectively. The experiments show that the algorithm proposed in this paper has a faster convergence speed of parameter optimization, has a higher fault-diagnosis accuracy, is more sensitive to small samples, and achieves the best fault-diagnosis effect compared to the traditional algorithm, realizing a 98.93% correct fault-identification rate.

## Figures and Tables

**Table 1:** Fault classes for the four-op-amp biquadratic high-pass filter.

Fault Name	Incipient Fault Class	Nominal Value	Fault Value
F0	NF	/	/
F1	C1	5 nF	3 nF
F2	C2	5 nF	3 nF
F3	R1	6.2 kΩ	7.44 kΩ
F4	R2	6.2 kΩ	7.44 kΩ
F5	R3	6.2 kΩ	7.44 kΩ
F6	R4	1.6 kΩ	1.92 kΩ

**Table 2:** Parameter configurations for different models.

	Filters in the First Convolutional Layer	Filters in the Second Convolutional Layer	Kernel Size	Learning Rate	Dropout Rate
ATFCNN	64	128	5	0.01	0.4
GEO-ATFCNN	61	112	3	0.007011	0.0739
ECWGEO-ATFCNN	40	137	2	0.008407	0.3313

**Table 3:** Performances of different parameter-optimization algorithms.

Algorithm	ECWGEO	GEO	PSO	QPSO	WOA
Best	0.12925	0.20311	0.17731	0.21147	0.20938
Mean	0.13051	0.22158	0.22573	0.24894	0.22715
Worst	0.13177	0.24004	0.24400	0.31562	0.24389

**Table 4:** Performance of different models.

Algorithm	ATFCNN	CNN	BPNN	RNN
Best	98.93%	88.21%	90.71%	88.57%
Mean	98.45%	87.38%	87.98%	87.02%
Worst	97.86%	86.79%	85.36%	86.07%

## Data Availability

The raw/processed data cannot be shared at this time. Due to the nature of this research, participants of this study did not agree for their data to be shared publicly.
